# Uncertainty Compensation in Human Attention: Evidence from Response Times and Fixation Durations

**DOI:** 10.1371/journal.pone.0011461

**Published:** 2010-07-07

**Authors:** S. Lee Hong, Melissa R. Beck

**Affiliations:** 1 Department of Kinesiology, Indiana University, Bloomington, Indiana, United States of America; 2 Department of Psychology, Louisiana State University, Baton Rouge, Louisiana, United States of America; University of New South Wales, Australia

## Abstract

**Background:**

Uncertainty and predictability have remained at the center of the study of human attention. Yet, studies have only examined whether response times (RT) or fixations were longer or shorter under levels of stimulus uncertainty. To date, no study has examined patterns of stimuli and responses through a unifying framework of uncertainty.

**Methodology/Principal Findings:**

We asked 29 college students to generate repeated responses to a continuous series of visual stimuli presented on a computer monitor. Subjects produced these responses by pressing on a keypad as soon a target was detected (regardless of position) while the durations of their visual fixations were recorded. We manipulated the level of stimulus uncertainty in space and time by changing the number of potential stimulus locations and time intervals between stimulus presentations. To allow the analyses to be conducted using uncertainty as common description of stimulus and response we calculated the entropy of the RT and fixation durations. We tested the hypothesis of uncertainty compensation across space and time by fitting the RT and fixation duration entropy values to a quadratic surface. The quadratic surface accounted for 80% of the variance in the entropy values of both RT and fixation durations. RT entropy increased as a function of spatial and temporal uncertainty of the stimulus, alongside a symmetric, compensatory decrease in the entropy of fixation durations as the level of spatial and temporal uncertainty of the stimuli was increased.

**Conclusions/Significance:**

Our results demonstrate that greater uncertainty in the stimulus leads to greater uncertainty in the response, and that the effects of spatial and temporal uncertainties are compensatory. We also observed compensatory relationship across the entropies of fixation duration and RT, suggesting that a more predictable visual search strategy leads to more uncertain response patterns and *vice versa*.

## Introduction

Responses times (RT) have been a predominant measure of cognitive processing for over one hundred years [1], [2]. RT has often been used to make inferences regarding the amount of information processing, i.e., the level of difficulty of cognitive processing that must occur to process stimuli and make a response [3], [4]. For example, in a simple target detection task requiring subjects to press a button when a visual stimulus appears, RT is the time between when the stimulus appears and when the button is pressed. RT increases when the stimulus appears in an unexpected (i.e., uncued) location suggesting that the process of allocating attention is more difficult when there is spatial uncertainty [5], [6]. Similarly, Klemmer [7] demonstrated that RT increased with greater “temporal uncertainty” due to longer pre-stimulus intervals.

Although uncertainty and predictability have remained at the center of the study of human attention, uncertainty has been used only to describe the pattern of the stimulus presentations, and not the responses. As a result, there has been relatively little work that has used uncertainty as a description of both stimulus (i.e., independent variable) **AND** response (i.e., dependent variable) characteristics within a unifying framework of uncertainty. Therefore, the current study is designed to examine whether spatial and temporal uncertainty in the stimulus begets greater uncertainty in the response. Further, we seek to examine whether stimulus uncertainty has similar effects on the response (RT) and search (eye movement) components of attention.

The first goal of the current research is to examine the influence of stimuli uncertainty on the amount of uncertainty in the distribution of RTs in a target detection task that requires sustained attention. Contrary to conventional studies of attention that involve only the measurement of mean RT, the current study employs measures uncertainty of the response patterns by analyzing the distribution of RT data. One reason for using the distribution instead of mean RT is that the key assumptions needed for the valid use of mean RT are not always met. The key assumptions for mean RT are: 1) the RTs possess a normal distribution; 2) their variance is random; and 3) this reflects the idealized case of situations where only correct responses are produced. While one can argue that insuring the accuracy of the responses is fundamentally necessary, the more recent literature brings into question the former assumptions. First, rather than satisfying assumptions of a normal distribution, RTs have been shown to be the superposition of a normal and exponential distribution [8]. Although Heathcote et al. [8] have developed the Ex-Gaussian method specifically for non-normally distributed RT data, the Ex-Gaussian approach does not provide a direct measure of uncertainty contained within the RTs. Furthermore, in studies where repeated responses to stimuli (akin to continuous performance tasks) were conducted, it has become evident that the sequence of RT is not random [9], [10]. Thus, a second reason for using the RT distribution in our current study is that if the response sequence is not completely random, consisting of a unique pattern known as the 1/*f* process [9], [10], there is the potential that the shape of the distribution of RTs in itself has information to offer. As a result, our first question is to ask whether the uncertainty contained within the distribution of responses changes as a function of stimulus uncertainty.

The second goal of the current study is to examine the influence of different sources of stimulus uncertainty (spatial and temporal) on the distribution of responses. There is now a significant portion of the literature showing that lower spatial uncertainty leads to shorter RT [6], [11], [12]. In general, providing the subject with pre-cues of where the target stimulus will appear allows attention to be allocated to the target location more quickly resulting in shorter RT. Similarly, RTs are shorter when the uncertainty of the stimulus onset time is lower [13]–[17]. As with spatial uncertainty, reducing temporal uncertainty through pre-cues, for example, shows that temporal uncertainty alters the manner in which individuals orient their attention and results in changes to RT.

Whether spatial and temporal stimulus uncertainties have independent or interactive effects remains an issue of debate. There is literature based on brain activity to suggest that spatial and temporal attention activate different brain regions [14], [15]. Coull and Nobre [13] on the other hand, have argued for the interdependence of spatial and temporal stimulus uncertainties due to similarities in regions of brain activation. Furthermore, beyond the interactive vs. additive effects of spatial and temporal uncertainty on RTs, the manner in which it affects the distribution of RT data remains unknown. This leads to the second research question that seeks to determine how spatial and temporal stimulus uncertainties interact to alter the distribution of RT data (i.e., response uncertainty).

The third goal of the current study is to examine the effects of stimulus uncertainty on two different measures of attention, RT and fixation duration. Stimulus uncertainty may affect not only the distribution of RT but also the search behavior that occurs while waiting for the onset of a stimulus. Attention and eye movements have been tightly linked [18]–[21] indicating that eye movements can be used as a measure of the distribution of attention during the search component of a target detection task. Linking eye movement distribution patterns to that of RT could provide insight into the manner in which visual “input” is acquired in the lead up to the response (or “output”). It has been shown that subjects employing fewer fixations of longer duration during a visual search task had shorter RTs when compared to those who used more fixations of shorter duration [22]. This suggests that conducting a visual search with fixations that are on average longer and fewer in number lead to quicker responses. However, the distribution of human eye movements is also not random, and has been shown to exhibit a similar 1/*f* distribution [23], much like RT data [9], [10]. The third research question is whether the distribution of fixation durations follows that of the RTs and is similarly affected by spatial and temporal stimulus uncertainty.

For this study, we use Information Entropy [24] as a means of quantifying the distributions of the data, which also is a measure of uncertainty within a data set [25]. There are three major benefits to the use of entropy (see [26] for a more in depth discussion), because entropy: 1) does not make assumptions regarding the data distribution (e.g., normality, random variance); 2) can be made conditional upon the satisfaction of certain criteria; and 3) can be linked theoretically to concepts of information processing and uncertainty [3], [4], [27]. Furthermore, entropy has been used previously to categorize changes in motor patterns as a function of uncertainty in the informational content from the environment [28]–[33].

The literature on human motor control that has involved entropy as a measure of the level of uncertainty has shown that muscle force output is lower when there is less information (i.e., greater uncertainty) contained in the visual feedback of the force output. This finding holds for both spatial [29]–[32] and temporal [28], [29], [33] uncertainties in visual feedback. These findings provided the basis for the *uncertainty compensation hypothesis*, where uncertainty is compensated across task, organism, and environment [26], [29], [34], [35]. As a result, we will conduct a preliminary test of whether this conceptual framework with uncertainty at its center can be extended to an attention task, and will be evident in the distributions of RTs and fixation durations.

While the combined effects of spatial and temporal uncertainty on RT remain a matter of debate, these uncertainties have been shown to have a compensatory effect on muscle force output. Hong et al. [29] have demonstrated that as the likelihood of obtaining visual feedback in space and time, the uncertainty contained in the force output decreased, reflective of fewer corrections being made to the force generated by the muscles. A majority of the variance in the data could be captured by a quadratic surface that showed that the muscle force patterns were similar when the spatial uncertainty of the visual feedback was high and temporal uncertainty was low and vice versa. It is an open question as to whether this pattern of change will hold for the fixation durations or RT.

Specifically, we use a modified version of the continuous performance task [36] to test the following research questions:

Q1. Does greater stimulus uncertainty lead to greater uncertainty in the distribution of the RT data?

Q2. How do spatial and temporal stimulus uncertainties affect the uncertainty contained in the distribution of RTs?

Q3. How do spatial and temporal stimulus uncertainties affect the uncertainty contained in the distribution of eye fixation durations?

**Figure 1:**
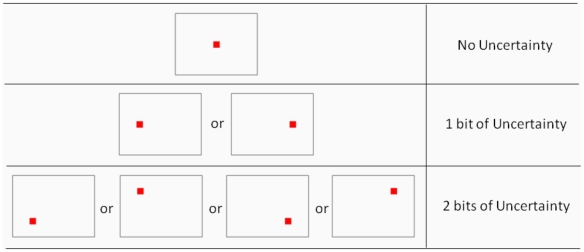
Illustration of the 3 different spatial uncertainty conditions. When there is no uncertainty, the stimulus always appears in the center of the screen.

The continuous performance task required subjects to press a button as soon as a red square appeared on a blank screen. We manipulated spatial uncertainty by changing the number of possible locations where the stimulus could appear (Figure 1). Temporal uncertainty was manipulated by varying the number of different inter-trial intervals (ITI) passed between each response and the onset of the next stimulus. RT was measured as the time between when a stimulus appeared and when a button was pressed and fixation durations were recorded using an eye movement tracker during the ITI.

Using entropy as a common measure of uncertainty contained in the distribution of RT and fixation durations, we seek to test whether the uncertainty compensation hypothesis in human motor control [26] holds in a visual attention task. It has been demonstrated that attention and eye movements share a common neural network [37], suggesting that the RT and eye fixation duration data should at least share some common properties. We thus employ the quadratic surface used in previous research [26], [34], [35], [38], [39] as a means of capturing the combined effects of spatial and temporal stimulus uncertainty on RT and fixation durations during a sustained attention task.

## Results

**Figure 2:**
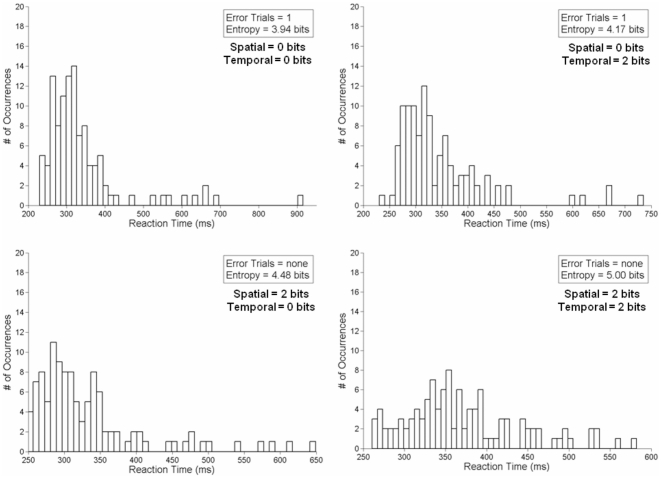
Exemplar probability distributions of RT data. These data were taken from a single subject under the 4 extreme stimulus uncertainty conditions. In clockwise order, we present the 0 bits – 0 bits, 0 bits – 2 bits, 2 bits – 0 bits, and 2 bits – 2 bits spatial and temporal uncertainty conditions, in a clockwise sequence, beginning from the top-left. Error trials denote situations where the subject either generated an anticipatory response (<100ms) or no response at all (>2000ms). The entropy values have been calculated using Eq. 1.

**Figure 3:**
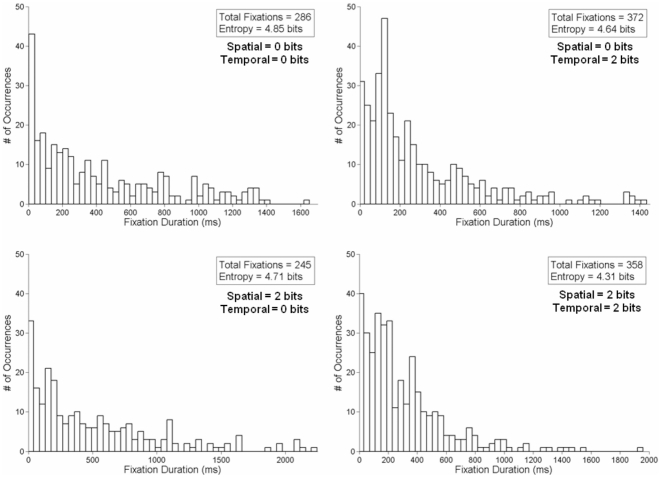
Exemplar probability distributions of fixation duration data. These data are from the same subject and conditions in Figure 2. Here, the total number of fixations within each trial block is presented alongside the entropy values, calculated using Eq. 1.

Exemplar entropy results from a single subject in the most extreme spatial and temporal stimulus uncertainty conditions (0 bits – 0 bits, 0 bits – 2 bits, 2 bits – 0 bits, and 2 bits – 2 bits, in a clockwise pattern) are provided in Figures 2 (RT) and 3 (Fixation Duration). In Figure 2, the general increase in entropy of the RT as a function of spatial and temporal uncertainty can be observed. This also illustrates of the broadening of the probability distribution as a function of increasing stimulus uncertainty. Figure 3 illustrates the changes in the entropy of the fixation durations as a function of the unpredictability of the stimulus. Here, the narrowing of the probability distribution can be observed, as a significant proportion of the data are clustered around the shorter fixation durations. What is also apparent in Figure 3 is that the changes in entropy are not necessitated by an increase in the number of fixations employed during a given trial block.

**Figure 4:**
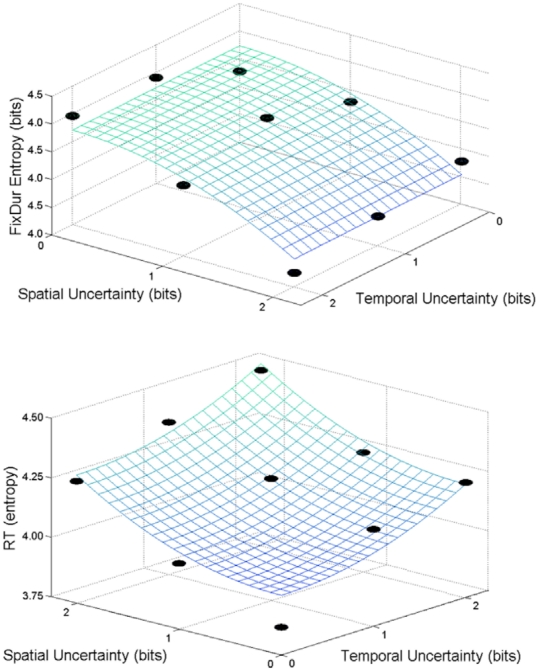
Results of the least-squares regression. Quadratic surfaces generated from the coefficients obtained from the least-squares regressions (Eq. 2 and 3), where eye fixation duration and RT entropy are presented as a function of the spatial and temporal uncertainty of the visual stimulus. Upper panel presents the surface obtained from the least squares fit to the eye fixation duration entropy data. Lower panel presents the results of the least squares fit to the RT entropy data.

Figure 4 shows the entropy values for fixation duration (upper panel) and RT (lower panel) as quadratic surfaces with parameter values obtained from the least-squares fit. For the entropy of the fixation durations, all of the parameters of the regression model were statistically significant (*p*<.05), with the exception of the *a_space_* parameter (see Table 1), and the model was able to account for 80% of the total variance.

**Table 1:** Regression results from the least-squares fit of Eq. 2 to the eye fixation duration data.

Source of Variance	*df*	*SS*	*MSE*	*F*	*p*	Variance Accounted
Model	2	0.093	0.047	49.44	<.001	80.5%
Unexplained	6	0.023	0.004			
Total	8	0.116				
Parameter	Parameter Value		*t*		*p* (one-tailed)	
*k_eye_*	4.345		245.423		<.001	
*a_time_eye_*	0.001		0.080		.469	
*a_space_eye_*	0.060		3.377		.007	

**Figure 5:**
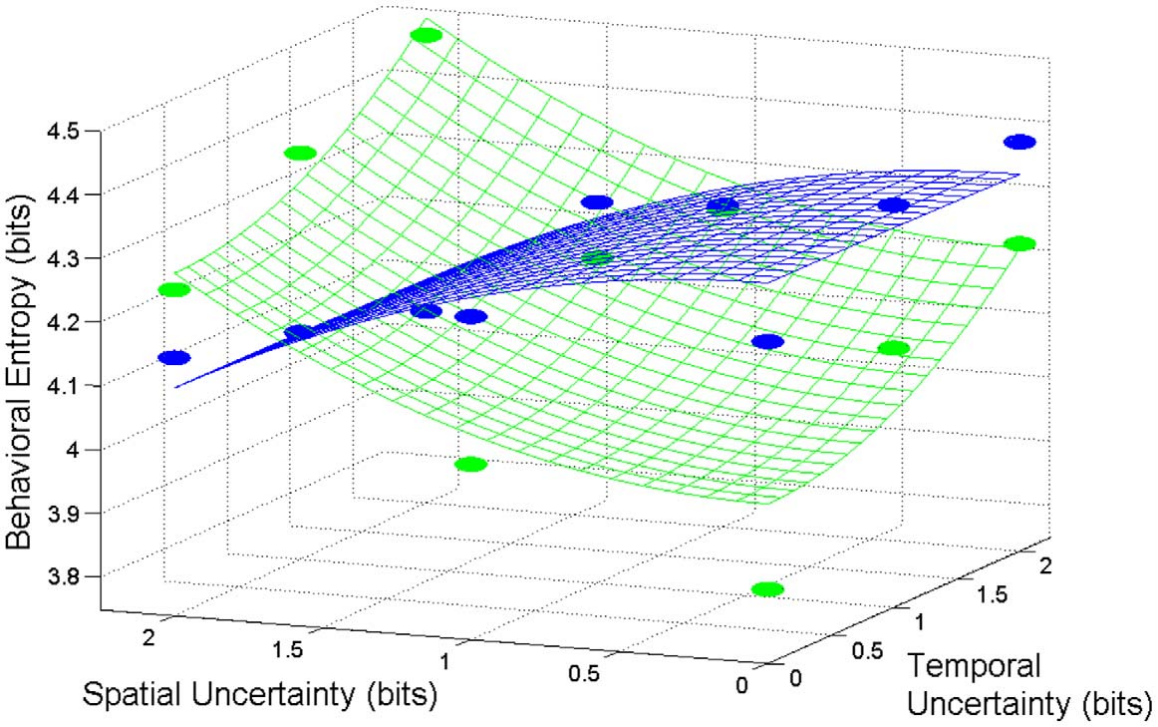
Combined quadratic surfaces for RT and fixation duration. The surfaces represent both cognitive and motor entropy as a function of spatial and temporal uncertainty of the visual stimulus. The blue surface and data points represent the eye fixation duration entropy data while the green surface and data points represent the RT entropy data.

Similar to the entropy of the eye fixation durations, the model of the RT data accounted for 80% of the total variance. For the RT entropy data, all of the parameters of the regression (see Table 2) model were statistically significant (*p*<.05). From both the upper and lower panels of Figure 4, it can be seen that 2 bits of spatial uncertainty coupled with 0 bits of temporal uncertainty in the stimulus led to an approximately similar RT entropy value as 0 bits of spatial uncertainty with 2 bits of temporal uncertainty. The models in Equations 2 and 3 can then be combined to form a single surface (Figure 5) that captures the stimulus-response relationship in terms of bits of uncertainty.

**Table 2:** Regression results from the least-squares fit of Eq. 3 to the RT entropy data.

Source of Variance	*df*	*SS*	*MSE*	*F*	*p*	Variance Accounted
Model	2	0.215	0.108	48.68	<.001	80.2%
Unexplained	6	0.053	0.009			
Total	8	0.268				
Parameter	Parameter Value		*t*		*p* (one-tailed)	
*k_RT_*	4.000		147.329		<.001	
*a_time_RT_*	0.056		2.067		.042	
*a_space_RT_*	0.072		2.638		.019	

## Discussion

The results of this study provide support for the uncertainty compensation hypothesis [26], [29], [34], [35], extending it to human attention. Specifically, the entropy of the RT and fixation durations changed in a compensatory manner to one another, where: 1) Information Entropy of RT increased as the spatial and temporal uncertainty of the stimuli increased; and 2) Information Entropy of the fixation durations decreased as the uncertainty of the stimuli increased. These results provide the following answers to the three research questions presented in the introduction.

Q1. Does greater stimulus uncertainty lead to greater uncertainty in the distribution of the RT data?

*Yes, uncertainty in the RTs increased as a nonlinear function of stimulus uncertainty.*

Q2. How do spatial and temporal stimulus uncertainties affect the uncertainty contained in the distribution of RTs?

*The effects of spatial and temporal stimulus uncertainties on the RT entropies were compensatory.*

Q3. How do spatial and temporal stimulus uncertainties affect the uncertainty contained in the distribution of eye fixation durations?

*There was a compensatory effect of spatial and temporal stimulus uncertainty on the entropy of eye fixation durations, which decreased as stimulus uncertainty increased.*

Beyond being qualitatively similar to previous research, the variance accounted by the quadratic surfaces is comparable to the 80% observed in a previous experiment [34]. The entropy of the RT opposed that of the fixation durations, with entropy increasing as a function of greater stimulus uncertainty. Interestingly, the quadratic surface used to describe uncertainty compensation hypothesis in motor behavior [26], [34], [35] captured the change in entropy of the fixation durations as a function of stimulus uncertainty. By simply reversing the signs of the equation in the quadratic surface from negative to positive, we were able to also apply the quadratic surface to the RT data. Thus, the model suggests that the increases in entropy of the RT as a function of greater stimulus uncertainty occurred in concert with a nearly mirror-symmetric decline in the entropy of the fixation durations (see Tables 1 and 2). This symmetric relation between the entropies of the RT and fixation durations is evidence of a potential compensatory relationship between them.

As the uncertainty in stimulus location and ITI increased, so did the entropy of the RT, suggesting that in terms of RT, stimulus uncertainty begets response uncertainty. Thus, as the stimulus presentation became more unpredictable, the breadth of the RT distribution increased (see Figure 2). The systematic change in RT entropy across the different conditions suggests that the pattern of RT variation is not random [9], [10] and is consistent with the proposal that cognitive variability may exist for adaptive purposes [40]. Results of the quadratic surface for RT entropy suggest compensatory effects of spatial and temporal stimulus uncertainty on response uncertainty. This is evident in the similar levels of RT entropy when spatial uncertainty was high and temporal uncertainty was low and *vice versa* (Figure 4 – lower panel).

The compensatory effect of increasing spatial and temporal uncertainties on the entropy of the fixation durations is reminiscent of our previous findings of entropy compensation across motor output and environment [29], [34], [35]. However, the lack of significance in *a*_time_ parameter for the fixation duration entropy suggests that temporal uncertainty did not significantly affect the entropy of fixation durations to the level of spatial uncertainty, despite the relative strength of the model fit overall. Perhaps, the difference of 500 ms between ITIs was not sufficiently large to elicit significant changes in the distribution of fixation durations (Figure 3). Nevertheless, the phenomenon of increased entropy in fixation durations is interesting, especially since the entropy of the fixation durations was lowest when temporal and spatial uncertainties were at their highest.

From a behavioral perspective, our results show that instead of increasing the breadth of the distribution of their fixation durations in an attempt to “search” for the stimulus, the subjects resorted to fixations of generally similar duration (lower entropy, narrower distribution). Effectively, this points to a situation where the subjects were taking “snapshots” of their visual environment at more consistent time intervals. This finding is similar to previous research that found subjects resorting to increasingly fewer bimanual coordination patterns [34] and corrections to muscle force output [29], [35] when the availability of visual information is reduced (greater uncertainty). Perhaps, the greater stimulus uncertainty prompts this increased regularity in fixation durations as a strategy to minimize the likelihood of “missing” the stimulus when it arises. In sum, the results show that the subjects adapted their visual search behavior to adjust to the different levels of stimulus uncertainty.

Overall, our data support the hypotheses of interdependencies between: 1) space and time stimulus uncertainty; and 2) search (fixations) and response (RT) components of attention. The interdependencies between space and time are consistent with the literature for both RT [13] and motor performance [41]. Our results show that the pattern of change in RT entropy can be modeled as the symmetric mirror opposite of the entropy of the fixation durations (see Figure 5), suggesting that the search and response components of attention are inherently linked. More importantly, it shows that the uncertainty across search and response possess a compensatory relationship, where a predictable search pattern leads to a more uncertain response pattern and *vice versa*.

At a more theoretical level, our findings support Fitts and Posner [27] hypothesis of constant channel capacity for a single task, revealed in relationship between stimulus and response uncertainty. Beyond increasing RT, we show here that greater uncertainty in the stimulus leads to greater uncertainty in the response. Effectively, our results suggest that the amount of uncertainty in the stimulus (i.e., amount of information to be processed prior to the response) is reflected in the response patterns, appearing as a systematic increase in entropy of the RT with greater stimulus uncertainty. The phenomenon of “entropy conservation” in stimulus-response patterns has been previously shown in the brain activity of animal models [42], which may be a neurobiological link between the current study and what Fitts and Posner [27] proposed as the fixed information processing channel capacity based on behavioral data. Overall, our study represents a promising first step in the application of an uncertainty-based framework to describe the patterns of stimuli, visual search strategy, and responses.

## Materials and Methods

### Subjects

Twenty-nine (6 male, 23 female) undergraduate students with a mean age of 19.4±1.4 (*SD*) years volunteered for this study. All subjects possessed normal or corrected-to-normal vision and were free of any neurological or neuromuscular disorders. This study was conducted according to the principles expressed in the Declaration of Helsinki. This study was approved by the Louisiana State University Institutional Review Board and all subjects provided written informed consent for the collection of samples and subsequent analysis prior to beginning the study.

### Apparatus

An SR EyeLink II eye tracker was used to present the stimuli, record the key press responses and the eye movement data. The EyeLink recorded eye movements at a sampling rate of 250 Hz and a spatial resolution of approximately 0.5° using an infrared video-based tracking technology to compute the center and size of the pupils in both eyes. The head was stabilized by means of a chin rest located 47 cm from the monitor. During each trial, the eye tracker goes off-line for 188 ms after a response is generated. As a result, we could not record eye-tracking data could for the first 188 ms of every inter-trial interval (ITI). The eye tracker was re-calibrated prior to the commencement of each block to account for subtle changes in head position. The EyeLink system also provided us with RT data for each trial.

### Continuous Performance/Response Task

Subjects were instructed to pay close attention to the computer monitor and respond as quickly as possible when a target appeared on-screen. The target was a 20×20 pixel red square measuring 0.8 cm and subtending a 0.98° visual angle at a viewing distance of 47 cm. The target was presented on a 19-inch CRT monitor set at a 1024×768 resolution with a 60 Hz refresh rate. Subjects were instructed to press either the left or right side button on the SR Eyelink button box (Microsoft Sidewinder gamepad) as soon as the target appeared on the screen, and were told that either press would suffice as a response. When a response was provided, ITI timing for the next target began immediately after the response. If the subject did not generate a response within two seconds of the onset of the target, the target was removed and the next ITI began. Subjects were provided with the opportunity to rest between trial blocks.

### Experimental Design and Instructions

Subjects completed 9 blocks of 112 trials for a total of 1,008 trials. However, *the first trial of each block had to be discarded as lag times at the start of the program were being added by the data collection software to the RT*. Thus, we were left with 111 trials per block. The level of spatial and temporal uncertainty was set for each block of trials. There were three levels of uncertainty for each variable (high, medium and no uncertainty), yielding a 3 (Time)×3 (Space) fully crossed, repeated-measures design (total of 9 different conditions).

These estimates of bits of uncertainty were conducted as per Hick [3] that is log_2_ (N), where N is the number of alternatives in an equal distribution. For the different levels of spatial uncertainty, the number of possible target locations was varied. There was one possible spatial location (no uncertainty), two possible spatial locations (1 bit of uncertainty), or four possible spatial locations (2 bits of uncertainty). In the no uncertainty spatial condition, the target always appeared in the center of the screen. In the 1 bit of uncertainty spatial condition, the target could appear at the center of the left or right side of the screen. Both possible locations were centered vertically and 6.8 cm (8.3°) to the left or right of the horizontal center of the screen. As a result, there was a distance of 16.6° visual angle from the center of the target to the center of the screen. In the spatial condition with 2 bits of uncertainty, the target could appear in one of the four corners of a 14.5 cm×9.2 cm invisible square centered on the screen. From the center of the target locations, there was a 16.6° horizontal visual angle and a 10.2° vertical visual angle between target locations. Each square was 9.7° from the center of the screen. These spatial conditions are represented in Figure 1. Within each level of spatial uncertainty, the target appeared in all locations with equal probability. The order of spatial location for a trial block was randomly ordered.

For the temporal variable, the ITI between targets varied. There was one ITI (no uncertainty: 1250 ms), two ITIs (1 bit of uncertainty: 1000 ms, 1500 ms), or four ITIs (2 bits of uncertainty: 500 ms, 1000 ms, 1500 ms, 2000 ms). Within each level of temporal uncertainty, all possible ITIs occurred with equal probability, insuring that the mean ITI for all trial blocks remained at a constant 1250 ms. The order of ITIs across trials was presented in a random order for each block.

### Preprocessing of data

Eye movement behavior can be categorized primarily as either fixations or saccades. In general, eye movements and attention have been shown to be tightly linked [18]–[21]. A saccade is defined as an eye movement with acceleration greater than 4000°/s^2^ and an angular velocity greater than 22°/s. A fixation is defined whenever acceleration and velocity are less than the aforementioned levels, as the eye is considered as being fixated on a given point in space. This allowed us to determine the duration and number of fixations during the ITI. If no response was generated within a 2 second period, the trial was considered an error trial. Similarly, to prevent the inclusion of potential anticipatory responses (especially in the low uncertainty conditions), trials where the RT was below 100 ms were also considered as error trials. The removal of the responses satisfying the aforementioned criteria resulted in the loss of only 1.4% of the data, indicating that, on average, the subjects were able to maintain a 98.6% level of accuracy. These errors were also observed to be generally evenly distributed across subjects and conditions.

### Estimation of Entropies for RT and Fixation Duration

The most critical aspect of the data analysis is to determine differences in the “shape” of the distribution that is independent of size or magnitude of the variance. Furthermore, the entropy analysis should be conducted so that it is not affected by the mean, allowing us to tease out the changes in “structure” of the variation in the RT and eye fixation data.

In the case of the RT data, the error trials have to be removed in order to prevent a heavy skewing of the data (due to superfast <100ms responses, and 2000ms non-responses). Here, we employ a previously used frequency histogram method of obtaining the probability distribution where *p_i_* represents the probability the occurrence of a data point within the *i* ^th^ bin [34]. The histograms were bounded between the minimum and maximum RT value for the block of trials, evenly divided into 50 bins. To maintain consistency across all conditions, regardless of error, *p_i_* were calculated as the number of data points within a given histogram bin divided by the total of 111 trials.

A similar approach was taken for the eye fixation duration data. However, without a one-to-one ratio between eye fixation and trials, the number of fixations differed from one block of trials to another. Thus, the histograms for the fixation durations had to be bounded between the minimum and maximum fixation duration value for the block of trials, and then evenly divided into *N*/10 bins, *N* being the total number of fixations throughout the block of 111 trials. Similar to the RT data, higher entropy values are indicative of a broader data distribution, while lower entropy values indicate a more peaked distribution.

Though minimal, error trials did occur, and we thus calculate the entropies of the RT as conditional entropy, to control for accuracy (in keeping with [3], [4], [43]), while removing the need for repeating blocks of trials that do not satisfy accuracy demands. Here, the probability that the task goal is achieved can be represented as the ratio of the number of correct responses to the total number of trials, represented as *p*_g_, must be introduced into the equation. The conditional entropy [25] of the eye fixation durations and RT, *H*, will then be based on the original probability distribution weighted by *p_g_*:
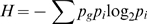
(1)Effectively, conditional entropies measure how much uncertainty is contained within the data distribution given that the constraint of response generation is satisfied.

Based on our previous experiments, we employ a sum of quadratic functions first presented in Newell, Liu, and Mayer-Kress [38] and also in our previous research [26], [34], [35], [39] to generate the least-squares regression. Here, the elevation of the surface represents the behavior while the horizontal dimensions of the surface provided a generalized description of the independent properties of the stimulus, i.e., the spatial and temporal uncertainty. The quadratic surface is a “bounded” function, i.e., converges to peak and minimum, and is thus preferred over the linear. To prevent “over-fitting” the data, we do not add linear components of the quadratic function into the fit (which was used previously [29]), since there are only 9 data points to fit (adding the linear components would mean that there would be only 5 free parameters used to capture 9 data points).

The sum of quadratic functions for the entropy of the fixation durations, *H_eye_*, takes the form:
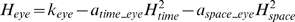
(2)Three free parameters, *k_eye_*, *a_time_eye_* and *a_space_eye_* will be obtained from a single least-squares regression. If the RT (cognitive) entropy, *H_RT_*, changes in a compensatory (mirror symmetric) manner to the entropy of the fixation durations, the signs would then be reversed, so that the sum of quadratic functions takes the form:
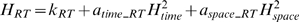
(3)
*H_time_* is the temporal entropy, and *H_space_* represents the spatial entropy of the stimulus, and *H_RT_* represents the entropy of the response times. Three free parameters, *k_RT_*, *a_time_RT_* and *a_space_RT_* will be obtained from a single least-squares regression. Across both models, *H_time_* and *H_space_*, are the independent variables of the spatial and temporal uncertainties of the stimulus.
